# A New Method of Preparing the Pure Gallic Acid

**Published:** 1804-03-01

**Authors:** M. L. Schnaubert


### A New Method of preparing the Pure Gallic Acid. By M. L. Schnaubert.

FOUR ounces of finely powdered galls are infused in water slightly alkalized with potash; the infusion which is of a very deep colour is filtred, and a solution of nitrate of tin is dropped into it, until no more precipitate is formed. The free acid of the liquid is then saturated with potash, taking care not to add the alkali in excess, and the precipitate is separated by filtration. The filtred liquid is then precipitated by acetite of lead, the weight and concentration of which is known. A greyish white precipitate is thus obtained, which is to be digested with diluted sulphuric acid, the proportion of the acid contained in which, must be to the concrete acetite of lead as one to four. After a sufficient digestion, the liquid is carefully filtred and evaporated, in order to afford crystals. If the gallic acid thus obtained, should contain a small quantity of sulphuric acid, it may be deprived of it by digestion with gallate of lead.

In order to be certain of the quantity of sulphuric acid necessary for the decomposition of a given quantity of gallate of lead, I previously dissolved four ounces of acetite of lead in sixteen ounces of water, and diluted one ounce of sulphuric acid in four ounces of water. By mixing these two liquids to afford the perfect precipitation, the requisite proportion was easily found. I must here observe, that it will be better to keep the gallic acid in a liquid state than to reduce it to crystals, because evaporation always renders it more or less brown. I am firmly persuaded that the gallic acid possesses the property of becoming brown by the action of light, at least, if in contact with the atmosphere. For if gallic acid be sublimed perfectly white, and then dissolved in water, it will be found after some time to have undergone this kind of alteration. M. Bucholt has made a similar remark relative to the same fact.

